# Multimodal Approach to Intraventricular Hemorrhage Using Echocardiography, Near-Infrared Spectroscopy, and Electrical Cardiometry in Preterm Infants

**DOI:** 10.21203/rs.3.rs-7376716/v1

**Published:** 2025-08-27

**Authors:** Aleksandra Hibner, Khang Tong, Lin Liu, Ana Morales, Shashank Sanjay, Henry Lee, Anup Katheria

**Affiliations:** UCSD; University of California, San Diego; University of California, San Diego; Sharp Mary Birch Hospital for Women & Newborns; University of California San Diego; Sharp Mary Birch Hospital for Women & Newborns

## Abstract

**OBJECTIVE:**

To investigate single versus combination hemodynamic parameters on intraventricular hemorrhage (IVH) or mortality in preterm infants.

**STUDY DESIGN:**

Data from 482 infants under 32 weeks gestational age were analyzed, including cerebral oximetry, mean arterial pressure (MAP), cardiac output, and systemic blood flow within the first 24 h. Wilcoxon Rank-Sum and chi-squared tests compared variables. Multivariable logistic regression and receiver operator curve (ROC) analyses assessed predictive value.

**RESULTS:**

Each additional gestational week was associated with lower odds of IVH (OR=0.66; 95% CI: 0.57–0.75) and mortality (OR=0.56; 95% CI: 0.45–0.69). Adjusted for covariates, right ventricular output (RVO) was associated with reduced IVH odds (AOR=0.996; 95% CI: 0.991–0.999), and higher MAP with reduced mortality (AOR=0.81; 95% CI: 0.68–0.94). Average NIRS <74% in 24 h increased mortality risk (OR=4.16; 95% CI: 1.46–11.0; P=0.005).

**CONCLUSION:**

Select hemodynamic measures are associated with IVH and death. Combining factors did not enhance early risk prediction.

## Introduction

Preterm infants, particularly those born at or before 32 weeks of gestational age, are at a significantly heightened risk of severe morbidities, including intraventricular hemorrhage (IVH) and death. (1) IVH is believed to result from multiple factors acting on the immature brain, with unstable cerebral blood flow being a significant contributor. (2) Impaired cerebral circulation is an early and prominent characteristic of the onset of IVH. These adverse outcomes are often linked to unstable hemodynamics during the critical early hours of life. The application of non-invasive modalities for improved prediction of IVH has been the subject of research for an extended period.

The use of echocardiography to assess cardiac function and systemic blood flow in preterm infants has been well described. (3–5) Studies have shown that echocardiographic parameters can be predictive of adverse outcomes, such as pulmonary hypertension and IVH. Kluckow et al have established that critically low superior vena cava (SVC) flow on echocardiograms is strongly associated with the development of IVH. (6–8) Additionally, other prospective observational studies have demonstrated that patients who developed IVH generally exhibited lower baseline left ventricular output (LVO) and right ventricular output (RVO), with a trend towards improvement prior to the onset of IVH. (9)

Near-infrared spectroscopy (NIRS) measuring cerebral tissue oxygen saturation (StO2) within the first 10 minutes of life was able to predict which infants would develop severe IVH and death. (10) Monitoring cardiac function and cerebral regional oxygen saturation (rSO2) can identify infants at higher risk for developing IVH before the onset of bleeding. Other noninvasive modalities studied extensively include electrical biosensing technology measuring cardiac output (CO). (11, 12) Although not a reliable standalone measure of CO in premature infants, it can be valuable for tracking changes in hemodynamic parameters over time, possibly when combined with other modalities. (13)

Computerized analysis of continuous blood pressure data in preterm infants has demonstrated that early postnatal hypotension—particularly prolonged periods of low mean arterial pressure—can predict adverse neurological outcomes such as severe IVH, ischemic cerebral lesions, and increased mortality. (14, 15) However, the predictive value of blood pressure alone is limited by this group's rapid hemodynamic fluctuations and immature cerebral autoregulation (16, 17), emphasizing the importance of combining continuous BP monitoring with additional methods like NIRS and functional echocardiography studies to better identify infants at risk for brain injury. (8, 18)

Clinical trials have already explored the use of multimodal hemodynamic monitoring in preterm infants, showing that combining various monitoring techniques can provide a more comprehensive assessment of an infant's hemodynamic status. (19) Limited evidence from studies, employing noninvasive monitoring techniques to predict and manage these hemodynamic abnormalities, has been inconclusive and has not demonstrated a clear benefit. (19, 20)

In this study, we aim to investigate whether a multimodal approach, combining various commonly used hemodynamic monitoring modalities, can improve the prediction of IVH and mortality in preterm infants. By integrating data from cerebral oximetry (StO2) measured by near-infrared spectroscopy (NIRS), mean arterial blood pressure (MAP), cardiac output (CO) measured by electrical cardiometry (EC), and systemic blood flow assessed via echocardiography (ECHO), we hypothesized a greater association with death or IVH with the combination of some or all of these modalities.

## Materials/ Subjects and Methods

This study was a retrospective cohort analysis of prospectively collected data from 5 randomized controlled trials. (10, 21–24) Institutional ethics board approval was obtained to conduct this secondary analysis. All data were taken from eligible participants admitted to the neonatal unit at Sharp Mary Birch Hospital for Women and Newborns in San Diego, CA, from January 2013 to December 2024 who had cardiovascular and hemodynamic monitoring data. Hemodynamic modalities included near-infrared spectroscopy (NIRS), mean arterial pressure (MAP), cardiac output (CO) using electrical cardiometry, and echocardiography (ECHO). Hospital records were examined, and head ultrasound images available on Synapse were assessed to determine the severity of IVH.

The near-infrared laser sensor (FORE-SIGHT Absolute Tissue Monitor, Casmed, Branford, CT) (25) was placed in the neonatal intensive care unit once the infant was considered stable by the medical team (1–3 hours of life). The sensor was positioned on the anterior forehead and standardized to minimize any effects on readings from different source positions. The Fore-Site Elite monitor has been calibrated against the INVOS device using a phantom model in the present analysis and using a neonatal probe (26), a threshold value of 67 was determined. Accordingly, a cutoff value of 67 was applied in our analysis to facilitate clinically relevant comparisons and interpretation.

Mean arterial blood pressure (MAP) was continuously measured through an indwelling umbilical arterial catheter (UAC) and recorded every 2 seconds. If the UAC was not in place, cuff blood pressure was obtained non-invasively and recorded every 2 to 6 hours.

**Figure 1:**
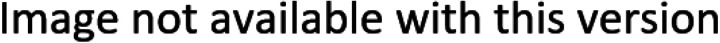
Figure legend not available with this version.

The electrocardiometry sensors (EC: Electrical Cardiometry, ICON device, Cardiotronic, La Jolla, CA, USA) were placed on the newborn’s chest, and stroke volume (SV) and cardiac output (CO) were recorded. Cerebral oxygen saturation, CO, and SV were recorded every 2 seconds and linked with recordings from the bedside monitor (heart rate, arterial oxygenation). All continuous variables were recorded for the first 24 hours. EC sensors were placed on the left side of the newborn's head (above the ear), over the left side of their neck, over the left axilla (at the level of the nipple), and the left inguinal canal (Fig. 1, Panel B). EC estimates cardiac output, stroke volume, and other hemodynamic parameters by sending a low-amplitude, high-frequency current through the body and measuring the resulting change in voltage across the thorax. This device can calculate changes in electrical conductivity to aortic blood flow at each contraction, enabling the calculation of stroke volume at each cardiac beat.(27, 28) We considered compromised cardiac output when values fell < 150 ml/kg/min.(28) All data were captured using a purpose-built digital data acquisition system (MP150, Biopac, Goleta CA).

A single functional echocardiogram was also performed in infants analyzed in the study within the first 12 hours of life using Vivid E9 (GE Healthcare, Wauwatosa, WI).

Measures of systemic blood flow, including the superior vena cava (SVC) and left and right ventricular output (LVO, RVO), were collected during each examination. A pediatric cardiologist reviewed all initial echocardiographic scans for structural abnormalities.

All the infants underwent transcranial ultrasound within the first 12 hours of life, again at 72 hours, and once more at 7 days of life. Radiologists blinded to the hemodynamic parameters interpreted the results, and Papile’s method was used to grade the diagnosis of IVH. (29)

## Data collection

Demographic and baseline data were collected, including gestational age, birth weight, antenatal steroid use, delivery method, sex, chorioamnionitis, and pregnancy-induced hypertension. The primary outcomes for this analysis were IVH severity and mortality. Since this was a convenience sample, an a priori sample size was not calculated. Relevant maternal and neonatal medical information was collected from the electronic medical records and recorded using the REDCap electronic database application hosted at SHARP Healthcare (REDCap v5.7.1 2014, Vanderbilt University). Additional variables comprised mean arterial pressure, cerebral NIRS readings, and cardiac output. All data were collected in the first 24 hours of life. The duration of cerebral hypoxia during which NIRS dropped below 67% and low cardiac output, when EC measurements fell under 150 ml/kg/min were extracted using Excel macros and Visual Basic for Applications (VBA). Echocardiography was conducted once within the first 24 hours of life to assess SVC, as well as both RVO and LVO.

## Statistical analysis

Continuous variables were summarized as mean and standard deviation (SD), while categorical variables were presented as counts and percentages. Variable summaries were stratified by three binary outcomes: any intraventricular hemorrhage (IVH), severe IVH, and death, and compared using Wilcoxon Rank-Sum and chi-squared tests for continuous and categorical variables, respectively. Univariable and multivariable logistic regressions were conducted to examine the association between potential predictors and outcomes. Multivariable analyses were conducted for two separate predictor sets: average/raw measurements set including mean over 24 hours (or raw value for echocardiograms) for MAP, NIRS, CO by electrical cardiometry, superior vena cava flow, RVO, and LVO; abnormal time set including total time over 24 hours with abnormal values (NIRS < 67, cardiac output < 150 ml/kg/min). Additionally, prenatal steroids and chorioamnionitis were considered as controlling variables for both analysis sets, while all analyses were controlled for GA. Birth weight was summarized but excluded from regression models due to collinearity with gestational age. The initial multivariable logistic regression model included potential predictors in each set with a univariable P-value < 0.20. Backward selection was used to iteratively remove the variable with the highest P-value and refit until all remaining variables had P < 0.10. This process was performed separately for any IVH, severe IVH, and death outcomes.

- In exploratory analyses, ROC curves were generated for each predictor to evaluate discriminatory ability for any-IVH and mortality. The optimal cutpoint for each predictor was determined using Youden’s index, area under the curve (AUC), the direction of association, sensitivity, specificity, positive predictive value (PPV), and negative predictive value (NPV) calculated. Predictors were then dichotomized at the optimal cutpoint, and their associations with each outcome were evaluated using univariable and multivariable logistic regression methods as described above to analyze these predictors on the original scale. All statistical analyses were performed using R Studio, version 4.3.0 (R Foundation for Statistical Computing), and a two-tailed P < 0.05 was considered statistically significant.

## RESULTS

Among the 482 infants born at < 32 weeks’ GA, 54 (11.2%) developed any-grade IVH, and 15 (3.1%) had severe IVH (grade 3 or 4) (Table 1). Baseline characteristics indicated that infants with IVH were significantly more preterm, had lower birth weights, and a higher incidence of chorioamnionitis compared to those without IVH (all P’s ≤ 0.001, Table 1). Severe IVH and mortality were associated with lower GA, MAP, and birth weights (all P’s < 0.01, Tables 2 and 3). GA was found to be strongly associated with each outcome, with each additional week of GA being associated with a 34% (OR = 0.66; 95% CI: 0.57–0.75), 42% (OR = 0.58; 95% CI: 0.44–0.76), and 44% (OR = 0.56; 95% CI: 0.45–0.69) reduction in the odds of any IVH, severe IVH, and mortality respectively (all P’s < 0.001). Unadjusted and adjusted analyses yielded consistent results for all three outcomes: any-grade IVH, severe IVH, and mortality.

Any-grade IVH: A 10 mL/kg/min increase in right ventricular output (RVO) was associated with a 4% reduction in the odds of any-grade IVH (AOR = 0.96 (0.92–0.99), *P* = 0.03). No variables from the time with abnormal measurements set were retained in the final models for any-grade IVH after adjustment for GA.

Severe IVH: After adjusting for GA, a 1 mL/kg/min increase in superior vena cava (SVC) flow was associated with a 4% reduction in the odds of severe IVH (AOR = 0.96 (95% CI: 0.93–0.99), *P* = 0.02) (Table 4). Each additional minute with abnormal NIRS (< 67%) was associated with a 0.2% increase in the odds of severe IVH (AOR = 1.002 (1.001–1.003), P = 0.004).

Mortality: Each 1 mmHg increase in MAP over the first 24 hours was associated with a 19% reduction in the odds of death (OR = 0.81; 95% CI: 0.68–0.94; *P* = 0.013) after adjusting for GA, SVC flow, and prenatal steroid exposure. No variables from the time with abnormal measurements set were significant predictors of mortality.

Hemodynamic parameters were analyzed collectively using a multivariable regression model. Gestational age emerged as a strong predictor of both outcomes, and after adjusting for it, other parameters did not contribute significantly to outcome prediction. In exploratory analyses, ROC curve analysis demonstrated that GA alone had the highest sensitivity and specificity for predicting both IVH and mortality (Tables 4 and 5). In a separate multivariable model using ROC-derived cut points, after controlling for GA, having an average NIRS value < 74% during the first 24 hours significantly increased the odds of mortality (OR = 4.16; 95% CI: 1.46–11.0; *P* = 0.005).

## DISCUSSION

In this extensive retrospective cohort of preterm infants born before 32 weeks’ gestational age, we investigated whether incorporating multimodal hemodynamic monitoring—including echocardiography, NIRS, MAP, and EC—could facilitate the earlier identification of infants at heightened risk for IVH and mortality. While parameters such as NIRS and SVC flow exhibited initial associations with lower IVH and mortality rates, these correlations diminished after adjusting for gestational age, indicating the predominant influence of gestational age in predicting outcomes.

Neonatologists increasingly recognize that assessing perfusion and tissue oxygen delivery requires more than blood pressure and oxygen saturation. (30)Prior research supports a multifactorial, physiology-based approach using contemporary monitoring technologies and individual patient profiles. The association between higher MAP and improved survival is biologically plausible given the dependence of cerebral perfusion on systemic BP in extremely preterm infants with immature autoregulation. Furthermore, treatments targeting hypotension did not enhance survival or neurodevelopmental outcomes and were associated with an increased risk of adverse events. (31)Our findings align with prior studies showing lower MAP in infants with IVH or who died, though we did not evaluate interventions or therapeutic choices based on BP values. (32) Recent literature cautions against relying solely on MAP, advocating for broader assessment including systolic and diastolic pressures and BP variability, which may better reflect cerebral hemodynamics. Jiang et al. identified a correlation between high BP variability and the resistance index of the anterior cerebral artery in a cohort of 92 infants, suggesting a link between IVH and these factors. (33)

Echocardiography remains valuable for detecting low-flow states. Prior studies have linked low SVC flow to higher IVH incidence, though our cohort did not show significant changes in LVO preceding IVH. (7, 9, 34)Emerging evidence suggests that early, targeted neonatal echocardiographic (TNE) assessment—when integrated into clinical decision-making—can improve outcomes in preterm infants by identifying hemodynamic instability and guiding timely interventions. (35)Studies have linked early TNE and hemodynamic screening with reduced mortality and lower rates of severe IVH, supporting the value of serial, physiology-guided echocardiography in optimizing care for this high-risk population. (36)NIRS values were not significantly different across groups, but secondary analysis suggested that sustained values below 74% may be associated with mortality. However, large trials like SafeBoosC-III have not demonstrated improved outcomes using NIRS-guided management. (37)A study by Alderliesten et al. found that mean arterial blood pressure (MABP) less than gestational age (in weeks) was not associated with lower cerebral oxygenation (StO2) or neurodevelopmental outcome scores. However, low StO_2_ was associated with lower neurodevelopmental outcome scores regardless of MABP. (38) While electrical cardiometry proved feasible for continuous monitoring, its weak association with IVH and mortality—consistent with prior studies—suggests limited prognostic utility. (13, 39–41)

Multimodal integration of modalities presents promising potential, especially in studies correlating cerebral oxygenation with echocardiographic and clinical parameters. (19, 20) Our results suggest that its added predictive value may be limited. The current reliance on BP alone is insufficient. Refining integrated, physiology-guided approaches and individualized assessments remains a valuable direction. Comprehensive monitoring is essential to improving outcomes in extremely preterm infants.

### Strengths and Limitations.

A key strength of our study is its substantial sample size and the use of prospectively collected physiological data from multiple randomized trials. This comprehensive hemodynamic approach, coupled with multimodal assessment, enhances both data quality and generalizability. Furthermore, the study employed time-resolved continuous data, such as second-by-second NIRS and electrocardiography (EC) readings, enabling detailed analysis of not only average values but also the duration of periods below clinically relevant thresholds.

However, several limitations should be acknowledged. Firstly, despite using prospectively collected data, the retrospective nature of the analysis may introduce bias from unmeasured confounders. Secondly, echocardiographic measurements were limited to a single time point within the first 12 hours, potentially missing critical fluctuations that could be relevant to the development of IVH. Thirdly, despite calibration efforts, variability between devices and operators in NIRS and EC readings may impact data accuracy. Lastly, while the study provided multimodal data, it did not evaluate true real-time integration, which involves simultaneous clinical decision-making based on multimodal trends. This aspect could be crucial in demonstrating a clinical advantage.

#### Clinical Implications and Future Research.

This study's findings have substantial implications for contemporary clinical practice and the future development of hemodynamic monitoring in the neonatal intensive care unit (NICU). Our findings corroborate GA as a pivotal factor in determining the risk of preterm infants. While individual hemodynamic parameters, such as RVO and SVC flow, provide valuable insights into cardiovascular status, as predictors of IVH or mortality when adjusting for GA. Ongoing prospective clinical trials such as the PIONIRS trial (NCT05708105) could advance research in premature infants through combined NIRS and echocardiography assessments. This could assist in guiding timely neuroprotective treatments and shaping future prevention strategies. Real-time, individualized hemodynamic stability assessment remains indispensable for guiding interventions, identifying evolving instability, and potentially mitigating secondary brain injury.

Future investigations should explore the potential of multimodal monitoring for outcome prediction, therapeutic guidance, monitoring response to interventions, and preventing secondary injury. Research into cost-effectiveness, workflow integration, and the impact on clinical outcomes will be critical to inform widespread adoption.

There should be a focus on dynamic, time-dependent modeling of hemodynamic changes rather than single-value cutoffs. Combining machine learning and real-time analytics with NICU bedside monitoring systems may facilitate more accurate risk stratification and better intervention timing. Moreover, randomized trials are imperative to verify whether clinical management based on multimodal hemodynamic feedback leads to improved outcomes.

## Conclusion

Routinely combining multiple hemodynamic parameters for early outcome prediction may not be justified in all preterm infants. Individualized, real-time monitoring and the integration of advanced analytics represent a promising strategy for improving outcomes in the most vulnerable neonatal populations but need further testing in larger randomized controlled trials.

## Supplementary Files

This is a list of supplementary files associated with this preprint. Click to download.

- SupplementaryTable1Hibner.xlsx
- SupplementaryTable2Hibner.xlsx
- Table1Hibner.xlsx
- Table2Hibner.xlsx
- Table3Hibner.xlsx
- Table4Hibner.xlsx
